# Odontogenic maxillary sinusitis and fungus ball development secondary to a dental root retained for more than 25 years. A case report

**DOI:** 10.4317/jced.59663

**Published:** 2022-06-01

**Authors:** Jordi Borrás-Ferreres, Miguel Armengot-Carceller, Cosme Gay-Escoda

**Affiliations:** 1 DDS, MS. Professor of the Master’s Degree Program in Oral Surgery and Orofacial Implantology, EFHRE International University/FUCSO, Barcelona (Spain); 2 MD, PhD. Head of the Department of Otorhinolaryngology, La Fe University and Polytechnic Hospital, Valencia (Spain). Professor of the Department of Surgery, Faculty of Medicine, Valencia University. BMCG IIS La Fe, CIBERES, Valencia (Spain); 3 MD, DDS, MS, PhD, EBOS, OMFS. Chairman and Professor of the Department of Oral and Maxillofacial Surgery, Faculty of Medicine and Health Sciences, School of Dentistry, University of Barcelona. Director of the Master’s Degree Program in Oral Surgery and Implantology, EFHRE International University/FUCSO, Barcelona. Coordinator/researcher of the IDIBELL Institute. Head of the Department of Oral and Maxillofacial Surgery and Implantology, Teknon Medical Center, Barcelona (Spain)

## Abstract

The displacement of a dental root fragment into the maxillary sinus is a serious complication of tooth extraction that can give rise to maxillary sinusitis. The condition can become chronic if the intrasinusal foreign body is not promptly removed, and Aspergillus fumigatus superinfection may ultimately result, forming a fungus ball. The present study describes the case of a 50-year-old man with fungal rhinosinusitis caused by the accidental displacement of a dental root over 25 years ago. The prolonged and atypical course of the disorder produced long diagnostic and therapeutic controversy, and justifies the publication of this clinical case, which affords useful information for routine clinical practice.

** Key words:**Odontogenic maxillary sinusitis, fungus ball, tooth displacement, aspergillosis, diagnostic and therapeutic controversy.

## Introduction

In 10-12% of all cases, maxillary sinusitis is of odontogenic origin, due to the intimate anatomical relationship between the teeth in the posterior zone of the upper jaw and the maxillary sinus (1). The causes of sinus infection of dental origin include apical abscesses, advanced periodontal disease and infected radicular cysts, while the iatrogenic causes include accidental introduction into the maxillary sinus of endodontic material or graft material used for sinus lift procedures, and tooth displacements into the sinus (2-4).

When sinusitis is caused by the presence of a foreign body within the sinus, mycotic infection may occur, fundamentally due to *Aspergillus fumigatus*, *Actinomyces* and *Nocardia* (5). The infection may be limited to the affected sinus (noninvasive presentations), giving rise to a chronic clinical condition that fails to respond to conventional treatment and tends to be mistaken for bacterial sinusitis, or it may invade neighboring structures (invasive presentations). In this case the disorder can be very serious and even prove fatal in immunocompromised patients – particularly when infection is due to certain fungal species of the genus *Rhizopus spp.* (6). Invasive infection caused by *Aspergillus fumigatus* may simulate carcinoma of the maxillary sinus, manifesting as a mass affecting the orbit, skin and bone, and spreading quickly towards intracranial structures. Treatment is based on the use of antifungals (fluconazole, voriconazole, etc.) and resection of the affected tissues, with a good prognosis. Serious presentations of this kind are most commonly reported in agricultural and farming scenarios (7).

Drug treatment for chronic maxillary sinusitis is often ineffective due to poor functioning of the ostium, which impedes the restoration of mucociliary flow and regeneration of the ciliary epithelium. In the case of localized mycotic chronic sinusitis or fungus ball, surgical treatment is curative and no antimycotic drug treatment is needed. In addition to removal of the mycotic and inflammatory lesions, adequate functioning of the maxillary ostium must be guaranteed (1,8,9).

The present study describes the clinical and radiographic evolution following the treatment of a case of odontogenic maxillary sinusitis with Aspergillus fumigatus superinfection, secondary to accidental displacement of a dental root over 25 years ago.

## Case Report

A 50-year-old man urgently visited the dentist for the evaluation of intense recurrent pain that seemed to originate in the right upper second molar. Specifically, the pain affected the posterior region of the right upper jaw and right temporal region. The clinical exploration revealed a 1.4-1.7 tooth-supported fixed partial denture that had been placed over 25 years ago, with a missing second premolar and first molar. The vitality and percussion tests proved normal. However, palpation of the sinus region and infraorbital foramen was very painful. The patient also described local pressure and fullness sensation. A diagnosis of acute maxillary sinusitis was suspected, and associated anterior and posterior rhinorrhea with a foul odor was confirmed, together with nasal respiratory insufficiency. The patient explained that he had suffered nasal obstruction, cacosmia and recurrent headaches on the right side for almost three decades. In fact, he had been diagnosed with “chronic migraine” by a number of physicians.

**Figure 1:**
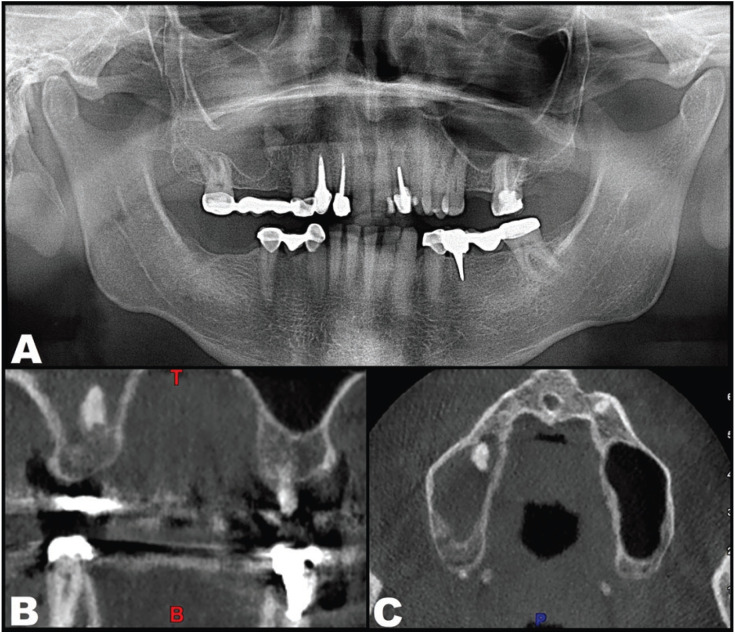
Initial radiographic findings. A. Panoramic X-ray view. B. Cone-beam computed tomography coronal view at the level of the root fragment displaced into the right maxillary sinus. C. Cone-beam computed tomography axial view at the level of the root fragment displaced into the right maxillary sinus.

The panoramic X-ray study showed complete opacification of the right maxillary sinus, with the presence of a foreign body. Cone-beam computed tomography in turn confirmed the presence of a dental root within the opaque sinus region, together with weak punctate mineralization close to the drainage ostium suggestive of a fungus ball (Fig. 1).

**Figure 2:**
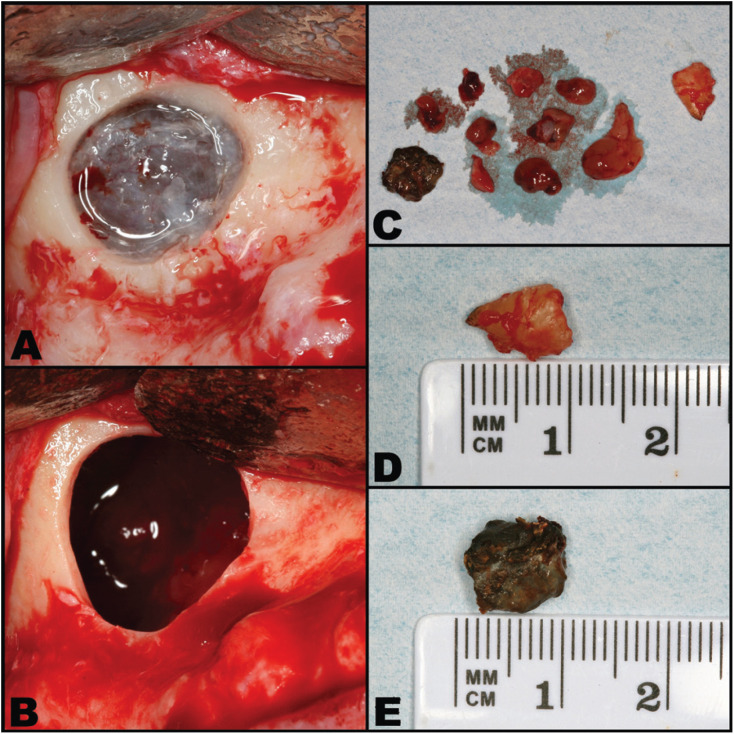
Modified Caldwell-Luc procedure. A. Exposure of the Schneiderian mucosa following ostectomy of the anteroexternal wall of the maxillary sinus. B. Exposure of the interior of the maxillary sinus after removal of the mucosa. C. Fragments of the edematous and hyperplastic sinus mucosa, with the fungus ball and root fragment. D. Detail of the dental root fragment. E. Detail of the fungus ball.

The patient accepted modified Caldwell-Luc surgery (without antrostomy of the inferior meatus) to remove the root fragment, the apparent fungus ball and the hyperplastic sinus mucosa. Although nasosinusal endoscopic surgery under general anesthesia was offered in the hospital setting, the patient preferred a maxillary approach under local anesthesia in the dental clinic. Extensive opening of the anteroexternal wall of the maxillary sinus was performed. After locating and extracting the dental root, curettes were used to remove all the hyperplastic and edematous mucosa lining the maxillary sinus. It was noted that the Valsalva maneuver did not result in air emerging from the buccosinusal communication, as the ostium was obstructed by the probable fungus ball. In order to remove the latter, the patient was instructed to repeat the maneuver several times until the pressure finally expelled a blackish fragment from the superomedial part of the maxillary sinus through the surgical window. After removing the fragment (which presented a moldy odor), air was seen to emerge on repeating the Valsalva maneuver – thus confirming correct ventilation of the maxillary sinus (Fig. 2). The histopathological study revealed large collections of *Aspergillus fumigatus* exhibiting an onion-peel distribution, consistent with aspergilloma secondary to a foreign body. The postoperative course was uneventful, with only moderate inflammation that fully resolved within 7 days.

**Figure 3:**
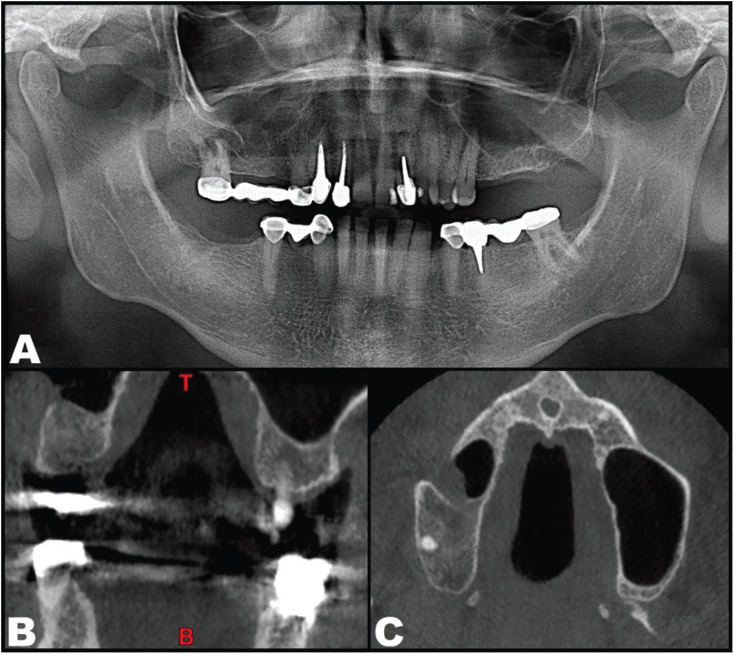
Radiographic control 12 months after surgery. A. Panoramic X-ray view. B. Cone-beam computed tomography coronal view. C. Cone-beam computed tomography axial view.

After 12 months of follow-up, all the clinical and radiological manifestations which the patient had suffered for over two decades had disappeared (Fig. 3).

## Discussion

Approximately 10-12% of all cases of maxillary sinusitis are of odontogenic origin, though the most recent literature suggests that the growth in implant surgery in recent years may be associated to an increase in the number of cases of iatrogenic sinusitis (1). In fact, a recent systematic review of 674 patients with odontogenic sinusitis found 65.7% of the cases to be of iatrogenic origin – the causes including accidental tooth, implant or dental material displacements, and buccosinusal fistulas (3). The estimated prevalence of the accidental displacement of upper posterior teeth into the maxillary sinus is 0.6-3.8% (4). When this problem occurs, most authors recommend surgical extraction of the foreign body in order to prevent sinus infection and its possible complications (4,10,11).

Although the underlying pathophysiology has not been fully clarified, it is believed that there is an important relationship between the fungal growth of *Aspergillus fumigatus* in the maxillary sinus and the presence of foreign bodies within the sinus (5,8,12). The presence of foreign bodies induces an inflammatory reaction that obstructs the ostium and alters ciliary function of the maxillary sinus – thereby favoring the growth of possible fungal spores carried in the inhaled air (3,5). This opportunistic fungal infection is characterized by noninvasive growth, with the development of an agglomeration known as a fungus ball (7,8). The literature contains multiple case reports describing the relationship between the appearance of fungus balls and the accidental displacement of dental materials, teeth and implants into the maxillary sinus (2,7,8,13). Most of the cases are asymptomatic, with nonspecific manifestations in the form of nasal obstruction, rhinorrhea, cacosmia, and facial or dental pain (5,8).

With regard to the clinical diagnosis, it is important to note that these cases manifest as unilateral chronic sinusitis refractory to usual medical treatment, and with an indolent course. Computed tomography is the exploratory technique of choice in these patients, characteristically revealing hypodense occupation of the maxillary sinus, which is seen to contain a hyperdense mass sometimes associated to microcalcifications. Over time, and in addition to the aforementioned findings, sclerosis may develop, with thickening of the bony walls or even focal bone erosions of the sinus wall (6).

In relation to the treatment of fungus balls, the fungal mass must be surgically removed from the sinus cavity, ensuring the restoration of correct ventilation and drainage of the maxillary sinus through its natural ostium (5). There is no need to prescribe systemic antifungal drugs, except in invasive presentations, in immune depressed individuals, or in the presence of complications (5,8,13).

With regard to surgery, the sublabial approach based on the modified Caldwell-Luc technique has been largely displaced by nasosinusal endoscopic surgery, and is now reserved for sinus neoplasms, traumatisms and for accessing the pterygopalatine and infratemporal fossa (9). Nevertheless, despite the success of the endoscopic technique, there are still some well documented indications for the Caldwell-Luc procedure, since it offers good access to the maxillary sinus and its adjacent structures. As commented above, the indications include intrasinusal tumors and cysts, foreign bodies, buccosinusal fistulas, maxillary osteonecrosis, fungus balls and facial injuries (9-11). Although fungal balls are ideally eliminated via nasosinusal endoscopic surgery, the buccal approach to the maxillary sinus through the canine fossa is also acceptable.

Maxillary sinus surgery based on the Caldwell-Luc approach conceptually contemplates an inferior meatotomy to favor sinus drainage. However, it is now known that this maneuver is neither necessary nor desirable, since mucociliary drainage is always directed towards the physiological ostium, and an inferior meatotomy interferes with the natural mucociliary flow (14,15). In addition, an inferior meatotomy may in some cases damage the nasolacrimal duct or the sphenopalatine artery (16). In relation to this, Huang *et al*. (17) published a retrospective study of 50 cases of odontogenic sinusitis treated using the sublabial Caldwell-Luc surgical approach without inferior meatotomy, and documented a more favorable postoperative course, due to the aforementioned reasons.

## Conclusions

The presence of foreign bodies within the maxillary sinus can give rise to chronic sinusitis which, if left untreated, may lead to fungal superinfection with the development of fungal balls. The torpid and indolent course of the condition, with nonspecific symptoms, complicates the diagnosis. The presence of prolonged unilateral manifestations refractory to the usual treatments, together with the almost pathognomonic radiological findings, guide the diagnosis. Treatment includes extraction of the foreign body and of the fungal agglomerates, based on nasosinusal endoscopic surgery or adopting the modified Caldwell-Luc approach. In any case, we also must restore correct ventilation and drainage of the maxillary sinus through its natural ostium. With the exception of very concrete cases, drug treatment for this mycosis is not indicated.
